# Supplementary material for: Comparative genomics provides new insights into the diversity, physiology, and sexuality of the only industrially exploited tremellomycete: Phaffia rhodozyma
Source: BMC Genomics. 2016 Nov 9;17:901. doi: 10.1186/s12864-016-3244-7 (PMC5103461; doi:10.1186/s12864-016-3244-7)
Supplement: Additional file 6: — List of orphan genes with links to PFAM (related to Additional file 1: Table S1). (ZIP 1428 kb) [file 12864_2016_3244_MOESM6_ESM.zip › PFAM_UNIQUE/G03963_P.html]

#                                                                            --- full sequence --- -------------- this domain -------------   hmm coord   ali coord   env coord
  
# target name        accession   tlen query name           accession   qlen   E-value  score  bias   #  of  c-Evalue  i-Evalue  score  bias  from    to  from    to  from    to  acc description of target
  
#------------------- ---------- ----- -------------------- ---------- ----- --------- ------ ----- --- --- --------- --------- ------ ----- ----- ----- ----- ----- ----- ----- ---- ---------------------
  
G03963\_P             -            977 DUF1033              PF06279.6    120       2.2    6.8   6.6   1   2   0.00012       0.7    8.4   0.0    70   100   100   129    79   139 0.78 - Protein of unknown function (DUF1033)  
G03963\_P             -            977 DUF1033              PF06279.6    120       2.2    6.8   6.6   2   2      0.29   1.8e+03   -2.6   4.2    25    55   453   483   448   490 0.79 - Protein of unknown function (DUF1033)  
G03963\_P             -            977 PyrI\_C               PF02748.10    52     0.014   13.5   2.5   1   2    0.0025       7.5    4.8   0.0    36    45    18    27    12    31 0.81 - Aspartate carbamoyltransferase regulatory chain, metal binding domain  
G03963\_P             -            977 PyrI\_C               PF02748.10    52     0.014   13.5   2.5   2   2   0.00031      0.93    7.7   0.7     5    50    67   115    65   117 0.81 - Aspartate carbamoyltransferase regulatory chain, metal binding domain  
G03963\_P             -            977 zf-FPG\_IleRS         PF06827.9     30      0.55    8.6   9.6   1   3     0.017        20    3.6   0.3    20    28    67    75    65    77 0.83 - Zinc finger found in FPG and IleRS  
G03963\_P             -            977 zf-FPG\_IleRS         PF06827.9     30      0.55    8.6   9.6   2   3     0.002       2.3    6.6   0.5    19    27   148   156   145   158 0.87 - Zinc finger found in FPG and IleRS  
G03963\_P             -            977 zf-FPG\_IleRS         PF06827.9     30      0.55    8.6   9.6   3   3     0.049        59    2.1   0.2     1     7   677   683   668   684 0.64 - Zinc finger found in FPG and IleRS
